# 42855 A Phenomics Approach to the Categorization and Refinement of Heart Failure

**DOI:** 10.1017/cts.2021.524

**Published:** 2021-03-30

**Authors:** Nosheen Reza, William Bone, Pankhuri Singhal, Anurag Verma, Ashwin C. Murthy, Srinivas Denduluri, Srinath Adusumalli, Macrylyn D. Ritchie, Thomas P. Cappola

**Affiliations:** Perelman School of Medicine at the University of Pennsylvania

## Abstract

ABSTRACT IMPACT: Measuring and analyzing qualitative and quantitative traits using phenomics approaches will yield previously unrecognized heart failure subphenotypes and has the potential to improve our knowledge of heart failure pathophysiology, identify novel biomarkers of disease, and guide the development of targeted therapeutics for heart failure. OBJECTIVES/GOALS: Current classification schemes fail to capture the broader pathophysiologic heterogeneity in heart failure. Phenomics offers a newer unbiased approach to identify subtypes of complex disease syndromes, like heart failure. The goal of this research is to use data-driven associations to redefine the classification of the heart failure syndrome. METHODS/STUDY POPULATION: We will identify < 10 subphenotypes of patients with heart failure using unsupervised machine learning approaches for dense multidimensional quantitative (i.e. demographics, comorbid conditions, physiologic measurements, clinical laboratory, imaging, and medication variables; disease diagnosis, procedure, and billing codes) and qualitative data extracted from an integrated health system electronic health record. The heart failure subphenotypes we identify from the integrated health system electronic health record will be replicated in other heart failure population datasets using unsupervised learning approaches. We will explore the potential to establish associations between identified subphenotypes and clinical outcomes (e.g. all-cause mortality, cardiovascular mortality). RESULTS/ANTICIPATED RESULTS: We expect to identify < 10 mutually exclusive phenogroups of patients with heart failure that have differential risk profiles and clinical trajectories. DISCUSSION/SIGNIFICANCE OF FINDINGS: We will attempt to derive and validate a data-driven unbiased approach to the categorization of novel phenogroups in heart failure. This has the potential to improve our knowledge of heart failure pathophysiology, identify novel biomarkers of disease, and guide the development of targeted therapeutics for heart failure.

